# American Agriculturist, for the Farm, Garden, and Household

**Published:** 1885-10

**Authors:** 


### American Agriculturist for the Farm, Garden and Household.

The American Agriculturist was established in 1842, and has been published continuously since that time. It has frequently been enlarged, so that now it is a quarto of 48 pages monthly. It has been so constantly improved, that it is difficult to conceive in what respects it could be made better. As an agricultural Journal it has no superior, if an equal in this, nor indeed so far as the writer is aware, in any other country.

This Journal is valuable and interesting not only to those engaged in agriculture, but to all classes of people—all are interested in agriculture whether they recognize it or not.

Horticulture and Floriculture also receive special attention.

Indeed a large range of subjects of general interest are presented and discussed. It is also fully and beautifully illustrated.

This is one of the few periodicals that ought to be in every family as an instrument of giving broad views and a generous culture, upon subjects that are too often neglected. It will serve a valuable purpose to the professional man as a diversion from the drudgery of his every day work. On many accounts we can strongly recommend the American Agriculturist. It is published at 751 Broadway, New York, at $1.50 per year. Any single number is worth far more than the price for a year.

